# The Influence of Slag Content on the Structure and Properties of the Interfacial Transition Zone of Ceramisite Lightweight Aggregate Concrete

**DOI:** 10.3390/ma17102229

**Published:** 2024-05-09

**Authors:** Haihong Fan, Shuaichen Chen, Rui Wu, Kaibo Wei

**Affiliations:** College of Materials Science and Engineering, Xi’an University of Architecture and Technology, Xi’an 710055, China; shuaichen@xauat.edu.cn (S.C.); 13572547200@163.com (R.W.); 18291387081@163.com (K.W.)

**Keywords:** interfacial transition zone (ITZ), ceramisite lightweight aggregate concrete, microstructure, pozzolanic reaction

## Abstract

Ceramisite lightweight concrete has excellent performance and relatively light self-weight characteristics. At the same time, the recent development of green high-performance concrete and prefabricated components has also brought the abundant utilization of these mineral mixture. An interfacial transition zone exists between the hardened cement paste and the aggregate, which is the weakest part of the concrete, characterized by high porosity and low strength. In order to study the effect of slag content on the interfacial transition zone in lightweight high-strength concrete, experiments were designed to replace cement with slag at different contents (0%, 5%, 10%, 15%). A series of studies was conducted on its macro-strength, microstructure, and composition. The results indicated that the addition of slag improved the porosity and width of the interfacial transition zone. Adding slag did not reduce the thickness of the concrete interfacial transition zone significantly at 3 d, but it led to significant improvement in the thickness of the interfacial transition zone at 28 d, and the thickness of the interfacial zone at 28 d was reduced from 19 μm to 8.5 μm, a reduction of 55%. The minimum value of microhardness in the slurry region of the interfacial specimens also increased from 19 MPa to 26 MPa, an increase of 36%. In addition, the structural density of the interfacial region was further increased, resulting in varying degrees of improvement in the macroscopic anti-splitting strength. One of the important reasons for this phenomenon is that the addition of slag optimizes the chemical composition of the interface and promotes the continuation of the pozzolanic reactivity, which further enhances the hydration at the interface edge.

## 1. Introduction

Ceramisite lightweight aggregate concrete has the characteristics of excellent performance and light weight. The bulk density of ceramisite aggregates is usually less than 1200 kg/m^3^, and the strength of ceramisite lightweight concrete is typically higher than 40 MPa, with a density of less than 2000 kg/m^3^ [1,2], making it more cost-effective. This construction method also improves efficiency, promoting its widespread application. In addition to its light weight, lightweight aggregate concrete also possesses the characteristics of good thermal insulation [3], excellent high-temperature resistance [4,5], good sound-insulation performance [6], and strong seismic performance [7]. With global sustainable development and China’s dual-carbon strategy in mind, there has been increasing research on the use of auxiliary cementitious materials, mainly mineral admixtures, to substitute cement in concrete [8]. When these mineral admixtures are used individually or in blends, they exhibit their respective properties during the hydration process of concrete, which affects their reaction to volcanic ash to varying degrees [9]. Further studying the mechanism of the effect of mineral admixtures on lightweight concrete will provide more references for the mix design of mineral admixtures [10,11,12,13].

Concrete microscopic modeling has been applied to categorize concrete into three components: the cementitious matrix, aggregate, and the concrete interfacial transition zone (ITZ) [14,15]. Compared with the cementitious matrix, the water–cement ratio in the interfacial transition zone is locally too high, resulting in a relatively high number of Ca(OH)_2_ crystals and ettringite in that zone, and a unilateral growth phenomenon occurs in the interfacial transition zone due to the adherence of Ca(OH)_2_ crystals to the surface of the aggregate [16,17]. Concrete deforms reversibly within a specific load range. However, once the limit of elastic deformation is surpassed, the deformation becomes irreversible. The porous structure and microcracks of ITZs often play a crucial role in determining the maximum load value. Therefore, ITZs are commonly considered the weakest part in construction works and concrete [18]. While lightweight aggregate concrete offers benefits such as reduced deadweight and excellent thermal insulation performance, its further development is constrained by the weakness of the ITZ. Therefore, the study of the ITZ holds significant importance.

In addition to unilateral growth effects, edge wall effects also occur between cementitious materials and aggregates [19]. There is a difference between the concentration of the cementitious material on the edge of the coarse aggregate and the actual slurry concentration, which results in different concentrations of various hydration products produced on the edge of the aggregate. This is also one of the reasons for the high porosity at the interface [20,21]. In addition to the edge wall effect, there are also micro-area drainage effects, unilateral growth effects [22,23], and dehydration shrinkage effects [24] during the formation process.

The addition of mineral admixtures to cementitious materials is believed to enhance the properties of cementitious composites [25]. Today’s research has made it possible to incorporate a wide range of materials with pozzolanic reactivity into cement [26]. Some examples of these materials include slag, silica fume, fly ash, and metakaolin [27,28,29]. It is particularly important to enhance the interface performance by incorporating mineral admixtures. The mixing of mineral admixtures in the cementitious materials can improve the interfacial bonding of concrete [30] and also reduce the width of the interfacial microcracks. However, excessive incorporation of mineral admixtures can also cause damage to the interface [31], resulting in a decrease in the strength of the concrete. In addition to the improvement of mineral admixtures for concrete interfaces based on cementitious materials, scholars have incorporated nanoceramics in cement to improve the properties of the interface, which in turn affects the carbonation properties of concrete [32] and has various effects on durability [33].

This article offers valuable insights into enhancing the interfacial transition zone (ITZ) of ceramisite lightweight concrete. It achieves this by examining ceramisite lightweight aggregate concrete produced using a variety of mineral admixtures combined with cementitious materials. Complemented by meticulous microscopic examinations, including electron microscopy and energy spectrum analysis, this study investigates the correlations between macroscopic strength, hydration products, pores, microcracks, ITZ thickness, and composition.

## 2. Materials and Methods

### 2.1. Materials

The cement used was P-O 42.5 ordinary Portland cement produced by Liquan Conch Cement Co. (Xianyang, China) and its performance is shown in Table 1; the fine aggregate was standard sand, conforming to the national standard, the coarse aggregate was ceramisite granule with a particle size of 5~10 mm, the water-reducing agent was polycarboxylic acid, the mixing water was tap water, the mineral admixture was slag, and the composition of the cement is shown in Table 2.

**Figure 1:**
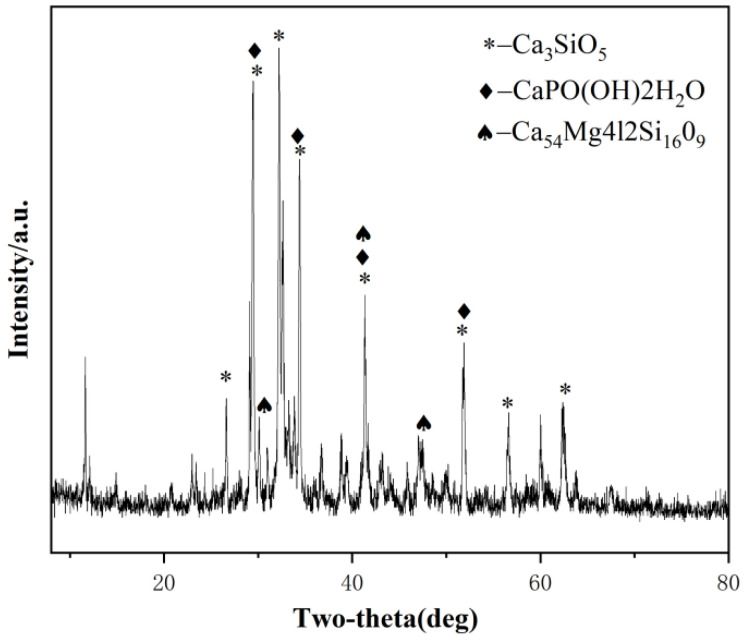
XRD patterns of slag.

The mineral composition of the slag was analyzed using X-ray diffraction (XRD), as shown in Figure 1. The instrument model used for the XRD test was the Rigaku SmartLab SE from Tokyo, Japan, with a copper target, a scanning angle of 5–90 degrees, and a scanning speed of 10 degrees per minute.

### 2.2. Proportioning Design

**Figure 2:**
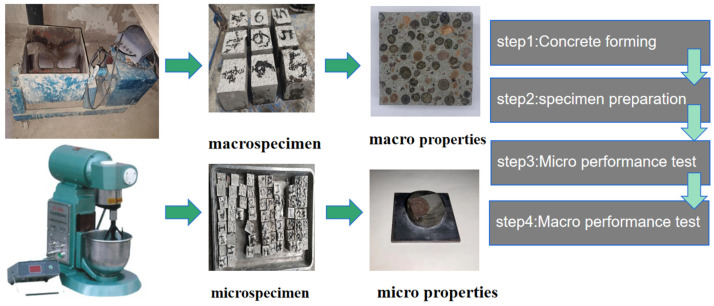
Flowchart of the process used in this work.

Table 3 provides the composition of all mix proportions. Mixes with 0%, 5%, 10%, and 15% replacements of cement mass with slag were examined. The concrete ratios and test method were according to JGJ/T12-2019 [34] (technical standard for application of lightweight aggregate concrete). The water–cement ratio was 0.30, which did not account for the water content in the coarse aggregate. Before mixing, the coarse aggregate was soaked in water for 24 h to ensure saturation. It was then dried on the surface with a towel and mixed in a mixer, followed by vibration and molding. After casting, the samples were left to cure at room temperature for 24 h. After demolding, curing was executed in standard atmospheric air at 22 ± 1 °C with a relative humidity of 90 ± 2% for 28 days. Three specimens of each proportion were prepared at each age for each ratio. An additional 3 specimens per proportion were required in case the experimental results of one group deviated too much and further testing was needed. The flowchart of this study is shown in Figure 2.

### 2.3. Sample Preparation

#### 2.3.1. Preparation of Samples for ITZ Observations

**Figure 3:**
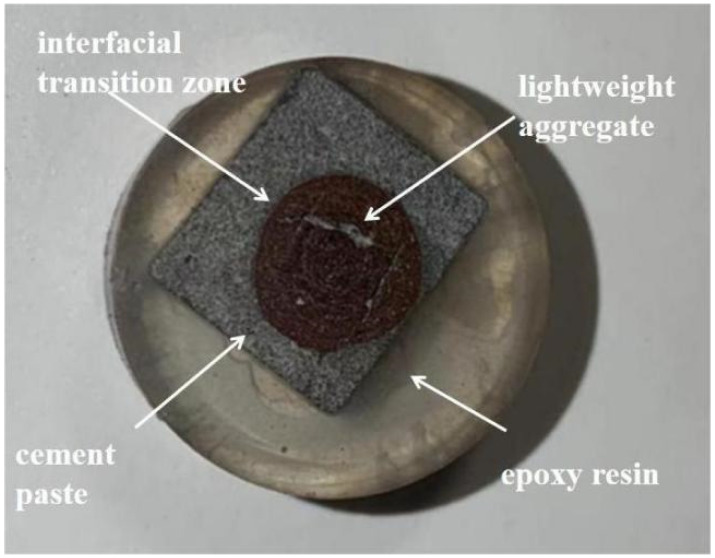
Interface sample.

Since it was difficult to observe the interfacial specimens of concrete, a mortar–aggregate mixture was prepared. Its components were weighed and mixed with water and admixtures in advance using a cement mortar mixer. Then, the mixture was poured into a 40 × 40 × 40 mm mold for vibration molding. After 24 h of storage at room temperature, the samples were demolded and then cured in a room temperature curing room. The samples were cured for 3 d, 7 d, and 28 d, respectively. To prevent excessive gaps in individual samples, we prepared three specimens of each age. Preventing too much inhomogeneity in one specimen, the samples were cut into cubic blocks measuring 10 × 10 × 10 mm using a cutting machine. Because we wanted to observe the interface specimens at different ages, we stopped the hydration operation for the specimens, immersed the cut specimens in anhydrous ethanol for 24 h, and dried them at 40 °C for 24 h in the drying oven. After that, we injected them with epoxy resin and dried them again at 40 °C for 24 h in a vacuum drying oven. Next, metallographic sandpaper of 400, 600, 800, 1000, 1200, 1500, 2000, and 3000 mesh was used for roughening, and finally, the samples were polished using a polishing flannel and polishing liquid. The fabricated specimen is shown in Figure 3.

#### 2.3.2. Specimen Preparation for Testing Macroscopic Properties

According to the GBT50081-2019 [35] Standard for Test Methods of Physical and Mechanical Properties of Concrete, 100 × 100 × 100 mm concrete specimens were prepared for the determination of splitting tensile strength.

### 2.4. Performance Testing

#### 2.4.1. SEM and EDS Testing

The samples to be tested were directly glued to the conductive adhesive and sprayed with gold for 45 s using an Oxford Quorum SC7620 (Quorum, London, England) sputtering coater at 10 mA; then, the samples were photographed using a ZEISS GeminiSEM 300 (Jena, Germany) scanning electron microscope for morphology and energy spectrum mapping, with an accelerating voltage of 3 kV for morphology photography and 15 kV for energy spectrum mapping. The accelerating voltage was 3 kV for topography and 15 kV for energy spectrum mapping, and the detector was a SE2 secondary electron detector.

#### 2.4.2. Microhardness Testing

**Figure 4:**
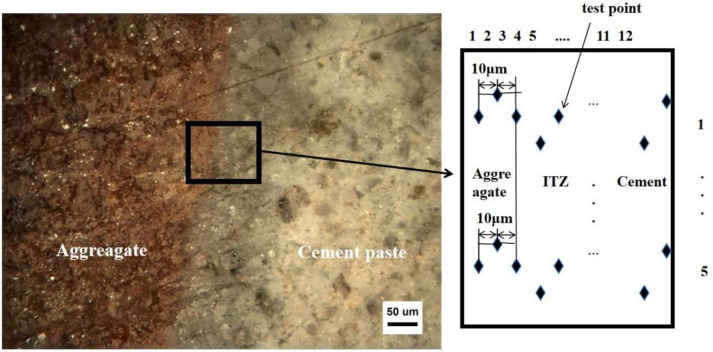
Microhardness test chart.

The microhardness test was performed using the HXD-10ZC/LCD (CANY, Shenzhen, China) Vickers hardness tester from the School of Materials Science and Engineering, Xi’an University of Architecture and Technology. The hardness measured when the applied loading force was less than 9.81 N is called microhardness and is usually expressed in Vickers (HV). The calculation formula is shown in Equation (1), and the test points and test images are shown in Figure 4.

(1) $$HV=0.102\frac{F}{S}\times \;2F\frac{\left(\frac{\theta }{2}\right)}{d^{2}}=0.1891\frac{F}{d^{2}}$$

In Equation (1), F—test load (N), S—surface area of the indentation (mm^2^):S = d′/(2sin(θ/2)):d—arithmetic mean value of the diagonal of the indentation (mm), θ—diamond indenter with a face angle of 136°.

#### 2.4.3. Macro Performance Testing

Bond-splitting tensile strength was used to characterize the macroscopic properties of concrete. The bond-splitting tensile strength at each time point was tested in accordance with the GBT50081-2019 Standard for Test Methods of Physical and Mechanical Properties of Concrete. The bond-splitting tensile strength experiment utilized the Xi’an University of Architecture and Technology Powder Research Institute TYA-2000 electro-hydraulic pressure tester (Xi’an, China).

## 3. Results and Discussion

### 3.1. ITZ Microstructure

**Figure 5:**
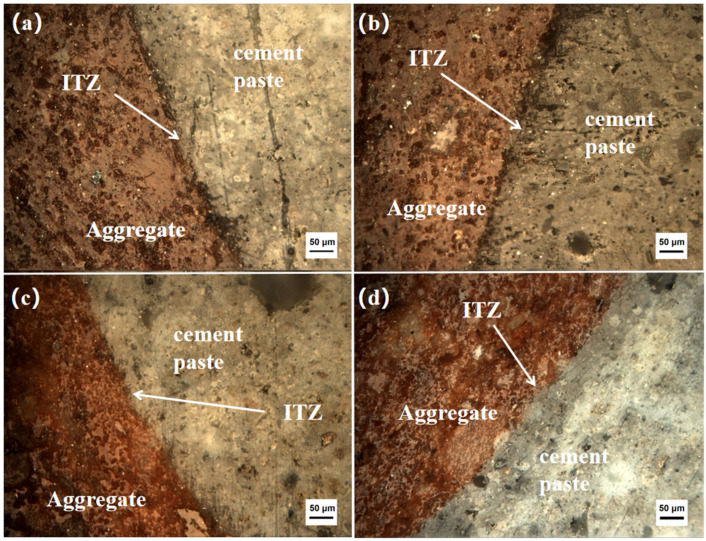
Optical microscope observation pictures of a specimen of each proportion under 28 d of maintenance: (**a**) interface specimen, group A0; (**b**) interface specimen, group A5; (**c**) interface specimen, group A10; (**d**) interface specimen, group A15.

**Figure 6:**
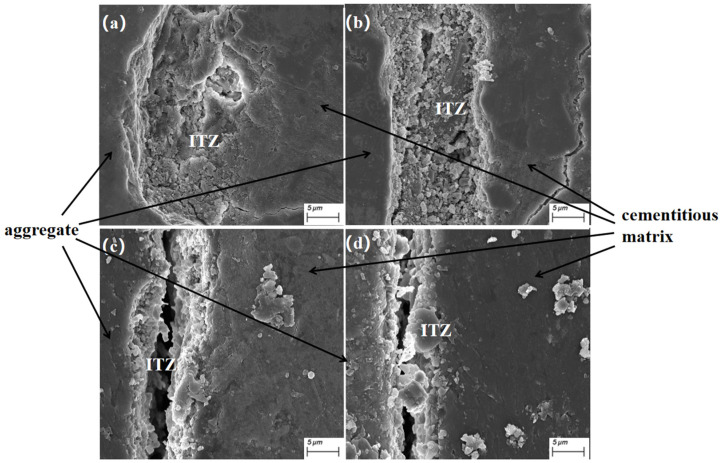
Scanning electron microscope images of a specimen of each proportion under 28 d conditioning: (**a**) interface specimen, group A0; (**b**) interface specimen, group A5; (**c**) interface specimen, group A10; (**d**) interface specimen, group A15.

Figure 5 shows the optical microscope photo of the concrete cured for 28 days; the distinct interface between the ceramsite and the cement paste is clearly visible. To examine the structure of the ITZ, SEM was utilized to capture photographs of the ITZ with various slag admixtures, as depicted in Figure 6.

As can be seen in Figure 5, the interface specimens made with different slag dosages did not show much difference in bonding under the optical microscope. However, there was a very obvious color difference between the aggregate and the cementitious matrix, as observed under the optical microscope. Nevertheless, it still remained difficult to observing the details and structure of the bonding area between the aggregate and cementitious matrix in the interface specimens.

The interfacial transition zone had different characteristics from both the cement paste and the ceramsite grains. The interfacial transition zone was significantly more porous and had features such as cracks, which were predominantly in the area where the paste was bonded to the aggregate and extend into the paste. This was where the fracturing of the aggregate and cement paste first began. The cracks and pores at the interface resulted in it being the weakest part of the concrete.

In Figure 6, the aggregate is shown on the left and the cement paste on the right. It is evident that as the slag admixture increased, more flaky particles could be seen at the edges of the interfacial zone. Based on the photographs, this appears to indicate an enrichment of hydrated calcium silicate at the interface. The presence of granular hydration products in the central part of the cracks appears consistent with the characteristics of calcium hydroxide (CH). With increasing slag admixture, the interface zone showed varying degrees of densification both in the interior and at the edges. When slag was not incorporated or when the amount of incorporation was low, obvious microporosity could be observed at the interface. Combined with the figure, we can see that in the case of curing for 28 d, the interfacial transition zone pores and microcracks of the concrete interfacial specimens without mineral admixture were the largest, with the ITZ width reaching 19 μm. For the 5% and 10% mineral admixtures, the ITZ widths were 11.5 μm and 9.5 μm, representing reductions of 44.7% and 50%, respectively, with respect to the cement-only group with no mineral admixture. The greatest interfacial improvement was observed with 15% slag doping, where the ITZ width came to 8.5 μm, a 55% reduction.

However, with the increase in the amount of slag, the microporosity at the interface nearly disappeared, which was also related to the reduction in the width of the interface. The density of the interface transition zone affects various properties of concrete [36,37].

### 3.2. Distribution of Elements in the ITZ

**Figure 7:**
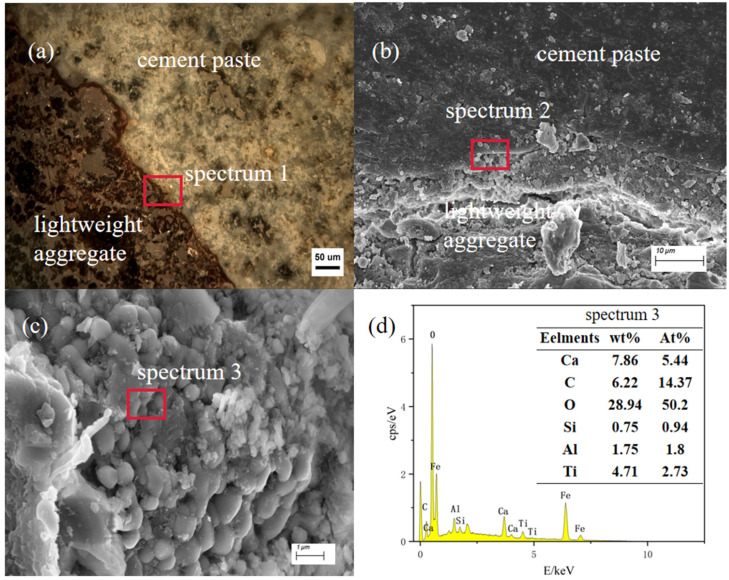
(**a**) Photomicrographs of ceramisite lightweight aggregate concrete, group A0 (unadulterated with mineral dopant); (**b**) ITZ photographs, group A0; (**c**) conformational photographs of ITZ spectrum2, group A0; (**d**) results of the energy spectral point sweep at spectrum3.

As indicated in the electron microscope images above, the interfacial transition zone exhibited a relatively loose structure and higher porosity compared with the cement slurry. Figure 6 depicts the photo of the boundary sample without any mineral admixture. Figure 7a represents an optical microscope image, while Figure 7b,c show electron microscope images at a higher magnification from the same observation position. Figure 7d presents the energy spectrum scanning results taken from the observation points in Figure 7c. The results clearly indicate that the boundary sample lacking mineral admixture possessed a wider thickness of ITZ. In Figure 7c, numerous granular hydration product phases were present within this interface transition zone. The spectrum 3 scanning results shown in Figure 7d reveal that there was a greater presence of C-S-H as the mineral phase in this area. Additionally, it can be observed that parts of the micropores were filled by the edge of the ITZ, gradually transitioning into the cement slurry.

**Figure 8:**
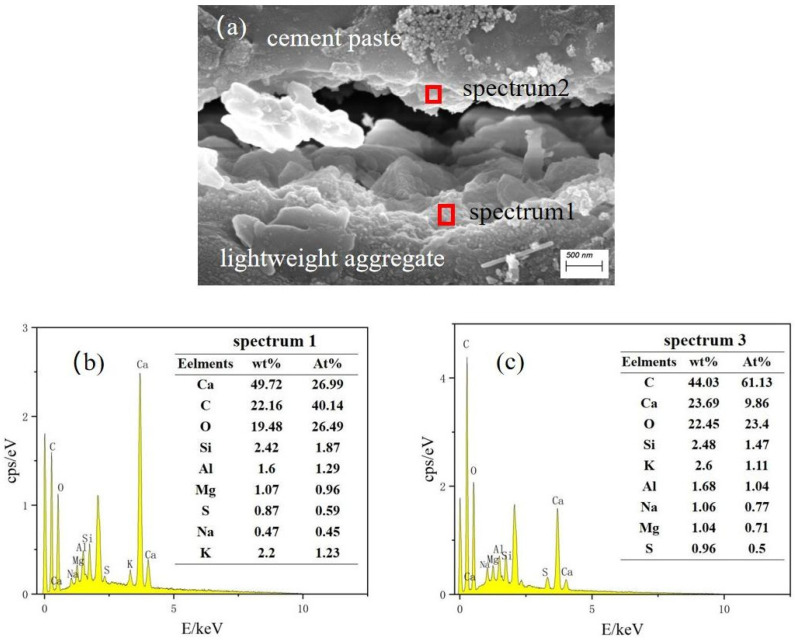
(**a**) Electron micrographs at the interface of ceramisite lightweight aggregate concrete, group A15 (mineral admixture of 15%); (**b**) results of energy spectrum spot sweep at spectrum1; (**c**) energy spectrum spot sweep at spectrum2.

Figure 8 shows the electron micrographs at the transition zone of the interface when the content of mineral admixture was 15%; spectrum 1 is the joint of the aggregate and cement paste. It is evident that flakes of AFm were produced at the interface, based on the electron micrograph morphology as well as the energy spectrum spot scanning results. AFm had a higher relative density than calcium alumina. However, after contact with SO_4_^2−^, it transformed into calcium alumina. This expansion led to an increase in volume, resulting in the generation of numerous gaps and microcracks at the interface joints, and a large amount of carbon (C) could be seen in spectrum2, which was due to the large number of capillary pores at the joints, where a part of the epoxy resin was adsorbed to fill part of the pores. The content of Ca, Si, Al, and other elements may also confirm that some ettringite (AFt) and AFm remained in the cement slurry after volume expansion.

Compared with ceramsite lightweight aggregate concrete without mineral admixtures, the width of the interface transition zone in ceramsite–cement paste with slag incorporation was smaller, and the structure was denser. The C-S-H existed at the edge of the interface transition zone, connecting the interface transition zone and the cement paste. The main substance exposed in the cracks in the central area of the interface transition zone was AFm. With the increase of mineral admixture, the Si content in the slurry increased, which expanded the C-S-H region and shortened the width of the interface transition zone, resulting in a denser structure. When slag was added, the calcium–silicon ratio decreased. The reaction between Ca(OH)_2_ generated by cement hydration and SiO_2_ in the cement paste reduced the enrichment level. The matrix contained more hydration products. Proper particle incorporation was beneficial for improving the performance of the interface transition zone [38].

### 3.3. Line Scan Results

**Figure 9:**
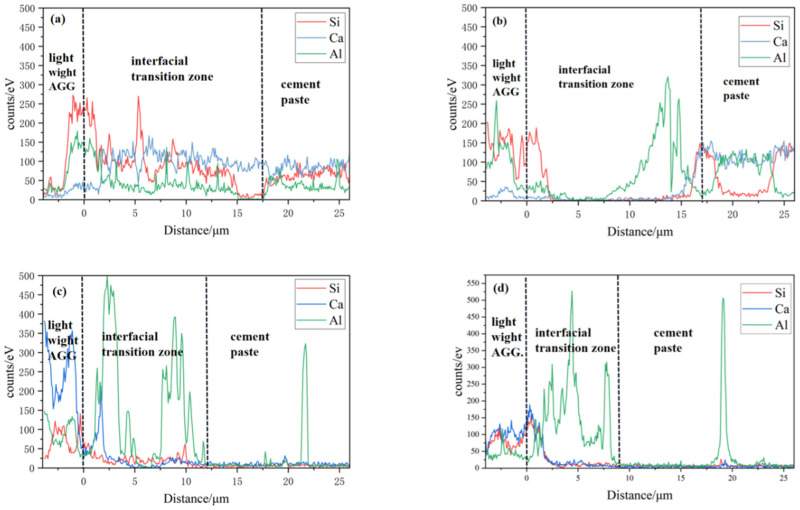
Line scans of the energy spectrometer for interface specimens of each proportion cured for 28 d: (**a**) interface specimen, group A0; (**b**) interface specimen, group A5; (**c**) interface specimen, group A10; (**d**) interface specimen, group A15.

The results of the line scan of the energy spectrometer for each element in the interfacial transition zone are shown in Figure 9, reflecting the distribution of Si, Ca, and Al elements in the aggregate–interfacial transition zone–paste region. Additionally, Al^3+^, Ca^2+^, and Si^4+^ in the cement paste also had different solubility, and they polymerized on the aggregate surface to form AFm and AFt, along with an oriented arrangement of crystals of Ca(OH)_2_. However, it is important to note that the strengths of all these hydration products were lower than that of the C-S-H produced by complete hydration.

The results show that there was only a small amount of Al in the cement paste, while the intensity peak of Al increased significantly at the interface transition zone. It was observed that Al appeared in large quantities at the edges of the cement paste with more pores and at the edges of the aggregate, accompanied by an elevation in O content. Microcracks were also seen in the polished interfaces, and with the incorporation of mineral dopants, the width of the microcracks and pores was reduced, to varying degrees. The migration of these elements was related to the concentrations of various ions within the cement. Previous studies have shown that cement paste in contact with the aggregate produces a sidewall effect, which is a major factor in the formation of the interfacial transition zone.

### 3.4. ITZ Thickness

**Figure 10:**
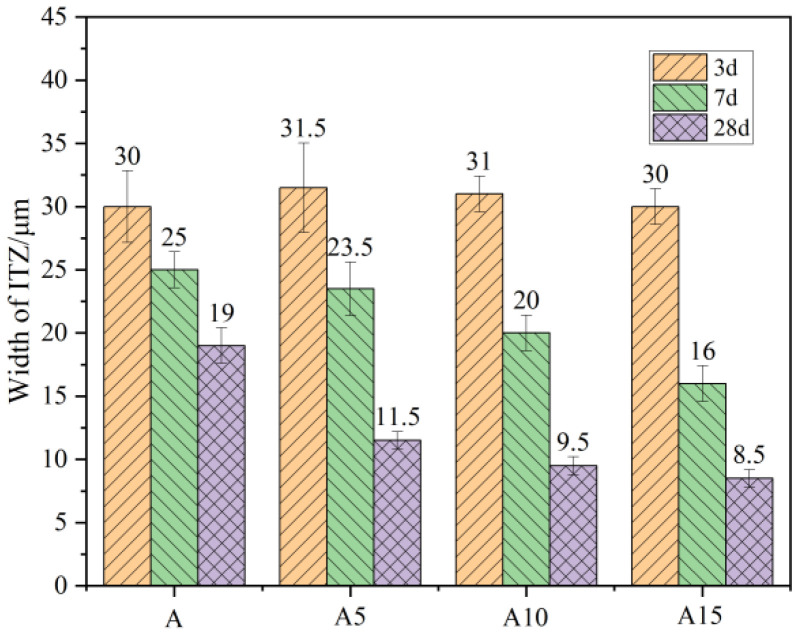
ITZ thickness of ceramisite lightweight aggregate concrete with different slag dosages.

The thickness of the ITZ was determined based on the distribution of elements described in Section 3.2. We used the change rate of Ca content as the basis for determining the boundary of the ITZ. If the change rate exceeded 50%, it was classified as the interface of the ITZ, and this helped us determine the thickness of the ITZ. This approach allowed us to obtain information on the thickness of the ITZ with different slag dosages. The thickness of the interface transition zone is shown in Figure 10.

Based on the results of the energy spectrometry, information related to the width and thickness of the interfacial transition zone was obtained. Figure 9 illustrates the variation in the thickness of the interfacial transition zone at different time points. Generally, the thickness of the interfacial transition zone decreased with the duration of concrete curing. Additionally, when the proportion of mineral admixture increased, the width of the interfacial transition zone at 28 d also showed a decreasing trend. The influence of slag incorporation on the width was not significant during the early stage of the hydration process. However, in the middle and late stages (28 d) of hydration, the width of the interfacial transition zone decreased by 36% with a 5% slag dosage compared with no addition.

**Figure 11:**
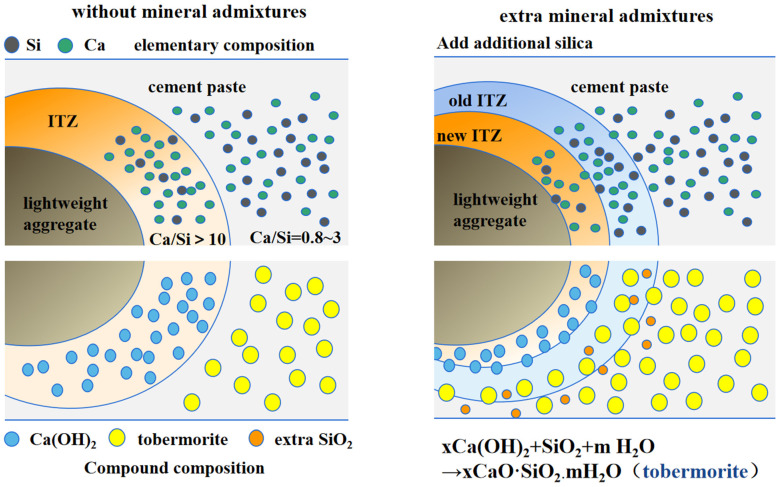
Mechanism of the effect of incorporation of mineral admixture on the transition zone with the interface.

At 15% content, the width of the interfacial transition zone was reduced by 52.9% to only 8.5 μm. From the width data of the interfacial transition zone and the electron microscope photographs, was confirmed that the width of the microcracks at the interface decreased and the bond in the interfacial transition zone became denser after the incorporation of mineral admixture [21]. Figure 11 reflects this process. This was attributed to the pozzolanic reactivity in the cementitious mix [39], where the Ca(OH)_2_ at the interface underwent the reaction described in Equation (2). This reaction allowed the Ca(OH)_2_ in the cement paste to participate in the reaction and promoted the transformation of Ca(OH)_2_ into C-S-H (tobermorite). The increase in SiO_2_ content in the mineral admixture further enhanced the hydration of the cement paste at the edge of the aggregate, resulting in a narrower interfacial zone.

xCa(OH)_2_ + SiO_2_ + mH_2_O → xCaO·SiO_2_·mH_2_O (tobermorite) (2)

### 3.5. ITZ Microhardness

**Figure 12:**
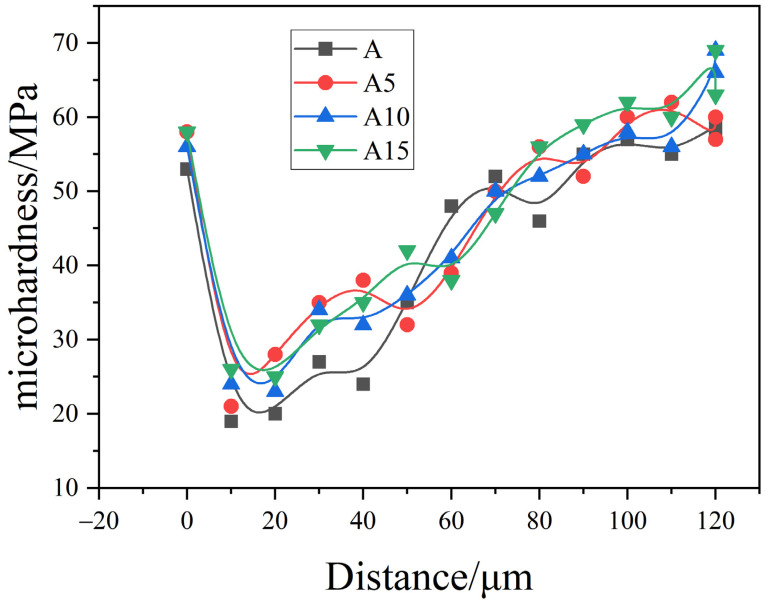
Microhardness of interface specimens with different slag dosages.

Figure 12 shows the microhardness test results for the interface specimens of each dosage of ceramisite lightweight aggregate concrete. The distribution pattern of the interface specimens with different dosages of mineral admixture was basically the same, and the microhardness distribution of the interface specimens existed in three obvious parts: the aggregate, the interfacial transition zone, and the mortar zone. The points between −20 μm and 0 μm on the interface specimen fell within the aggregate. There was a sharp drop in the microhardness value at 10 μm, which corresponded to the interfacial transition zone, and it gradually increased and stabilized as the range was extended to 100 μm. There was a difference in strength at the interface with different dosages. With the addition of slag, the strength at the interface at 10 μm was 19 MPa, 21 MPa, 24 MPa, and 26 MPa, respectively, as shown in the figure. The inclusion of mineral admixture made the structure at the interface denser and increased the microhardness, consistent with the microstructural characteristics described in the microstructure analysis.

### 3.6. Concrete Splitting Strength

**Figure 13:**
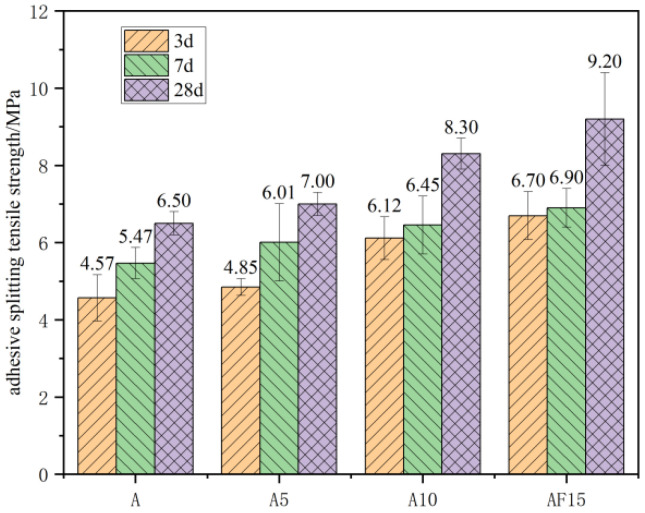
Split tensile strength of ceramisite lightweight aggregate concrete specimens with different slag dosages.

Figure 13 shows the splitting strength of ceramic stone lightweight aggregate concrete for each proportion, from which it can be seen that the bond-splitting strength of the concrete increased with the addition of mineral admixture. Considering the composition and structure of the interfacial transition zone as well as the bond-splitting strength, it can be concluded that the addition of mineral admixtures can improve the interfacial splitting strength by strengthening the interface itself. The thickness at the interface decreased with the increase in mineral admixture content, and the addition of slag densified the structure of the interfacial transition zone while reducing the porosity at the interface. This, in turn, led to a continuous increase in the splitting strength.

In summary, we can see that the porosity and structural densities of ITZ are directly related to the macroscopic strength of concrete, and the higher the strength and densities of ITZ, the better the mechanical properties of concrete. After modification of cementitious material, Hancheng Dan et al. [40] found that the microcracks in the interfacial transition zone were above 5 μm. Golewski, G.L. et al. [30] also investigated the effects of mineral admixtures of similar mineral composition on the interfacial zone. The width of microcracks was reduced by approximately 38%, 48%, and 30%, respectively, with blending [41].

## 4. Conclusions

In this paper, ceramisite lightweight aggregate concrete ratios were designed by replacing cement with different slag dosages. The structure and composition of the interfacial transition zones were analyzed using optical microscopy, scanning electron microscopy, energy spectroscopy, and microhardness experiments.

(1) The differences in light microscopic observations of the concrete interface specimens with different slag dosages were not significant. However, electron microscopic observations revealed that the porosity in the interface transition zone decreased and the structure became denser with increasing dosages of slag.
(2) The width of the interfacial transition zone was determined from the energy spectrometry results, and the composition and density of the elements at the interface were acquired via energy spectral line scanning; the thickness of the interfacial transition zone for each mineral admixture dosage was derived from the results, with the thickness at 3 d being around 30 μm, at 7 d being in the range 25–16 μm, and at 28 d being in the range 19–8 μm. Compared with the cement-only group, the width of the interfacial transition zone in the concrete with mineral admixture was reduced more significantly.
(3) Different slag dosages changed the formation of hydration products at the interface, with more Ca(OH)_2_, AFm, and AFt at the interface in the case of low dosages of slag. The addition of high-dosage slag resulted in more C-S-H at the interface, resulting in a denser and stronger interface.
(4) Slag admixture altered the mechanical properties of the interfacial zone and also enhanced the macroscopic strength of the concrete. In this study, it was found that slag incorporation improved the porosity and width of the interfacial transition zone, resulting in fewer pores, narrower width of the interfacial zone, and a denser structure. The interfacial transition zone properties of vitrified concrete are highly correlated with its macroscopic strength.

## Figures and Tables

**Table 1:** Cement performance indexes.

Density/(g·cm^−3^)	Specific Surface Area/(m^2^ kg^−1^)	Setting Time/min		Flexural Strength/MPa		Compressive Strength/MPa	
		Intial	Final	3 d	28 d	3 d	28 d
3.08	352	238	289	5.6	9.3	28.3	49.6

**Table 2:** Chemical composition of cement and slag.

Materials	Chemical Composition (wt.%)						
	SiO_2_	Al_2_O_3_	CaO	MgO	Fe_2_O_3_	TiO_2_	Other
slag	34.11	15.31	37.25	8.49	0.73	1.94	2.17
cement	22.53	7.22	57.04	2.89	3.53	0.34	6.45

**Table 3:** Mix proportion of concrete.

Number	Water/(kg·m^−3^)	Sand/(kg·m^−3^)	LWA/(kg·m^−3^)	Cement/(kg·m^−3^)	Slag	
					Desplacement Rate/%	Content/ (kg·m^−3^)
A	1440	4800	6700	4800	0	0
A5	1440	4800	6700	4680	5	120
A10	1440	4800	6700	4560	10	240
A15	1440	4800	6700	4440	15	360

## Data Availability

Data are contained within the article.

## References

[1] Wilson H.S., Malhotra V.M. (1988). Development of High Strength Lightweight Concrete for Structural Applications. Int. J. Cem. Compos. Lightweight Concr..

[2] Rossignolo J.A., Agnesini M.V.C., Morais J.A. (2003). Properties of High-Performance Lwac for Precast Structures with Brazilian Lightweight Aggregates. Cem. Concr. Compos..

[3] Nazari A., Sanjayan J.G. (2015). Synthesis of Geopolymer from Industrial Wastes. J. Clean Prod..

[4] Dong L., Yang Y., Liu Z., Ren Q., Li J., Zhang Y., Wu C. (2022). Microstructure and Mechanical Behaviour of 3D Printed Ultra-High Performance Concrete After Elevated Temperatures. Addit. Manuf..

[5] Dener M., Karatas M., Mohabbi M. (2021). High Temperature Resistance of Self Compacting Alkali Activated Slag/Portland Cement Composite Using Lightweight Aggregate. Constr. Build. Mater..

[6] Calleri C., Astolfi A., Shtrepi L., Prato A., Schiavi A., Zampini D., Volpatti G. (2019). Characterization of the Sound Insulation Properties of a Two-Layers Lightweight Concrete Innovative Façade. Appl. Acoust..

[7] Carrillo J. (2015). Effect of Lightweight and Low-Strength Concrete on Seismic Performance of Thin Lightly-Reinforced Shear Walls. Eng. Struct..

[8] Demirboğa R., Örüng İ., Gül R. (2001). Effects of Expanded Perlite Aggregate and Mineral Admixtures on the Compressive Strength of Low-Density Concretes. Cem. Concr. Res..

[9] Chen B., Liu J. (2008). Experimental Application of Mineral Admixtures in Lightweight Concrete with High Strength and Workability. Constr. Build. Mater..

[10] Zhang B., Poon C.S. (2015). Use of Furnace Bottom Ash for Producing Lightweight Aggregate Concrete with Thermal Insulation Properties. J. Clean Prod..

[11] Scrivener K., Martirena F., Bishnoi S., Maity S. (2018). Calcined Clay Limestone Cements (Lc3). Cem. Concr. Res..

[12] Tomar R., Kishore K., Singh Parihar H., Gupta N. (2021). A Comprehensive Study of Waste Coconut Shell Aggregate as Raw Material in Concrete. Mater. Today Proc..

[13] Parashar A.K., Gupta N., Kishore K., Nagar P.A. (2021). An Experimental Investigation on Mechanical Properties of Calcined Clay Concrete Embedded with Bacillus Subtilis. Mater. Today Proc..

[14] Khedmati M., Kim Y., Turner J.A., Alanazi H., Nguyen C. (2018). An Integrated Microstructural-Nanomechanical-Chemical Approach to Examine Material-Specific Characteristics of Cementitious Interphase Regions. Mater. Charact..

[15] Xie Y., Corr D.J., Jin F., Zhou H., Shah S.P. (2015). Experimental Study of the Interfacial Transition Zone (Itz) of Model Rock-Filled Concrete (Rfc). Cem. Concr. Compos..

[16] Lin J., Chen H., Zhang R., Liu L. (2019). Characterization of the Wall Effect of Concrete Via Random Packing of Polydispersed Superball-Shaped Aggregates. Mater. Charact..

[17] Scrivener K.L. (2004). The Interfacial Transition Zone (Itz) Between Cement Paste and Aggregate in Concrete. Interface Sci..

[18] Khedmat M. (2020). Multiscale Characterization to Examine the Effects of Aggregate Properties on Aggregate-Paste Interphase in Cement Concrete Mixtures. J. Mater. Civ. Eng..

[19] Diamond S. (2004). The Microstructure of Cement Paste and Concrete—A Visual Primer. Cem. Concr. Compos..

[20] Branch J.L., Epps R., Kosson D.S. (2018). The Impact of Carbonation on Bulk and Itz Porosity in Microconcrete Materials with Fly Ash Replacement. Cem. Concr. Res..

[21] Gao Y., De Schutter G., Ye G., Tan Z., Wu K. (2014). The Itz Microstructure, Thickness and Porosity in Blended Cementitious Composite: Effects of Curing Age, Water to Binder Ratio and Aggregate Content. Compos. Part B.

[22] Gartner E.M. (1997). A Proposed Mechanism for the Growth of C-S-H During the Hydration of Tricalcium Silicate. Cem. Concr. Res..

[23] YKe Y., Ortola S., Beaucour A.L., Dumontet H. (2010). Identification Microstructural Characteristics in Lightweight Aggregate Concretes Micromechanical Modelling Including the Interfacial Transition Zone (Itz). Cem Concr Res.

[24] Monteiro E.M.G.K. (2000). Proposed Mechanism of C-S-H Growth Tested by Soft X-Ray Microscopy. Cem. Concr. Res..

[25] Kishore K., Gupta N. (2021). Mechanical Characterization and Assessment of Composite Geopolymer Concrete. Mater. Today Proc..

[26] Lothenbach B. (2011). Supplementary Cementitious Materials. Cem. Concr. Res..

[27] Nežerka V., Bílý P., Hrbek V., Fládr J. (2019). Impact of Silica Fume, Fly Ash, and Metakaolin on the Thickness and Strength of the Itz in Concrete. Cem. Concr. Compos..

[28] Senff L., Hotza D., Repette W.L., Ferreira V.M., Labrincha J.A. (2010). Mortars with Nano-SiO_2_ and Micro-SiO_2_ Investigated by Experimental Design. Constr. Build. Mater..

[29] Assi L.N., Eddie Deaver E., Ziehl P. (2018). Effect of Source and Particle Size Distribution on the Mechanical and Microstructural Properties of Fly Ash-Based Geopolymer Concrete. Constr. Build. Mater..

[30] Golewski G.L. (2023). Concrete Composites Based on Quaternary Blended Cements with a Reduced Width of Initial Microcracks. Appl. Sci..

[31] Long Q., Zhao Y., Zhang B., Yang H., Luo Z., Li Z., Zhang G., Liu K. (2024). Interfacial Behavior of Slag, Fly Ash, and Red Mud-Based Geopolymer Mortar with Concrete Substrate: Mechanical Properties and Microstructure. Buildings.

[32] He H., Wang Y., Yuan J., Xu K., Wang S., Qiao H., Wu T., Yang J., Liu J., Yu J. (2023). A New Type of Mineral Admixture and Its Impact on the Carbonation Resistance of EPS Concrete. Sustainability.

[33] Sambucci M., Valente M., Nouri S.M., Chougan M., Ghaffar S.H. (2023). Enhanced Compatibility of Secondary Waste Carbon Fibers through Surface Activation via Nanoceramic Coating in Fiber-Reinforced Cement Mortars. Coatings.

[34] (2019). Technical Standard for Application of Lightweight Aggregate Concrete.

[35] (2019). Standard for Test Methods of Concrete Physical and Mechanical Properties.

[36] Golewski G.L., Sadowski T. (2016). Macroscopic Evaluation of Fracture Processes in Fly Ash Concrete. Sol. State. Phenom..

[37] Li X., Zhang Q. (2021). Influence Behavior of Phosphorus Slag and Fly Ash on the Interface Transition Zone in Concrete Prepared by Cement-Red Mud. J. Build. Eng..

[38] Golewski G.L. (2023). The Phenomenon of Cracking in Cement Concretes and Reinforced Concrete Structures: The Mechanism of Cracks Formation, Causes of Their Initiation, Types and Places of Occurrence, and Methods of Detection—A Review. Buildings.

[39] Berra M., Mangialardi T., Paolini A.E. (2018). Residual capability of alkali binding by hydrated pozzolanic cements in long-service concrete structures. Adv. Mater. Sci. Eng..

[40] Adam M.G.O., Koteng D.O., Thuo J.N., Matallah M. (2023). Effects of Acid Attack and Cassava Flour Dosage on the Interfacial Transition Zone Thickness, Durability and Mechanical Characteristics of High-Strength (Hs) Concrete. Results Eng..

[41] Golewski G.L. (2023). Mechanical properties and brittleness of concrete made by combined fly ash, silica fume and nanosilica with ordinary Portland cement. AIMS Mater. Sci..

